# Precision application of target-controlled infusion of esketamine combined with sufentanil in anesthesia for thoracoscopic surgery and its effect on hemodynamics, postoperative pain, and safety

**DOI:** 10.1590/1414-431X2025e15157

**Published:** 2026-03-02

**Authors:** Tianyu Qi, Xiangliu Liu, Sen Liu, Anqi Yin, Lidong Zhang

**Affiliations:** 1 Department of Anesthesiology, Jinling Hospital, Affiliated Hospital of Medical School, Nanjing University, Nanjing, Jiangsu Province, China

**Keywords:** Target-controlled infusion, Esketamine, Sufentanil, Thoracoscopic surgery, Hemodynamics

## Abstract

We aimed to expound the precise application of target-controlled infusion (TCI) of esketamine combined with sufentanil in anesthesia for video-assisted thoracoscopic surgery (VATS) and its effect on hemodynamics, postoperative pain, and safety. Eighty patients scheduled for thoracoscopic procedures were randomly assigned to either a control group [n=40, conventional empiric anesthesia (sufentanil plus propofol)] or an observation group [n=40, TCI of esketamine and sufentanil]. Hemodynamic indices [mean arterial pressure (MAP), heart rate (HR), central venous pressure (CVP), stroke volume (SV), cardiac output (CO), systemic vascular resistance (SVR), and oxygen saturation (SpO_2_)] were recorded before anesthesia (T0), after induction of anesthesia (T1), 30 min of anesthesia (T2), and at the end of surgery (T3). Recovery profiles [length of stay in the post-anesthesia care unit (PACU), awakening time], Ramsay sedation scores (T0-T3), visual analog scale (VAS) pain scores at 2, 24, and 48 h post-op, and adverse event rates were compared. The observation group showed smaller hemodynamic fluctuations from T1 to T3. At T3, this group had higher MAP, SV, and CO (P<0.05), steadier CVP and SVR, faster recovery (PACU stay and awakening times shorter, P<0.01), lower VAS scores at 24 and 48 h, higher Ramsay scores at T2 and T3, and lower overall adverse event rates (P=0.018) than the control group. TCI of esketamine plus sufentanil improved intraoperative hemodynamic stability, shortened recovery, enhanced early analgesia, and reduced adverse reactions in VATS, supporting its precision and safety.

## Introduction

Video-assisted thoracoscopic surgery (VATS) is a minimally invasive alternative to thoracotomy and has become a standard approach for diagnosing and treating thoracic diseases due to its advantages of smaller incisions, less postoperative pain, and quicker recovery (1,2). However, the unique challenges of thoracoscopic procedures - including one-lung ventilation (3) and carbon dioxide insufflation (4) - can significantly impact intraoperative hemodynamic stability and anesthetic depth control. These factors make the choice of anesthesia technique and agents critically important to ensure adequate sedation, analgesia, and circulatory stability throughout the procedure.

As a potent synthetic opioid, sufentanil is widely used in general anesthesia for its rapid onset and strong analgesic properties (5). Nonetheless, its use alone in VATS may result in respiratory depression, delayed emergence, or inadequate sedation depth (6). On the other hand, esketamine, the S(+)-isomer of ketamine, offers distinct pharmacodynamic advantages, including rapid induction, minimal respiratory suppression, and potent analgesia (7). Compared to racemic ketamine, it exhibits a 3-4 times greater affinity for NMDA receptors with less pronounced effects on respiratory and circulatory function (8). Notably, esketamine exhibits sympathomimetic effects, which may help mitigate the hypotensive episodes often seen with conventional anesthetic agents (9). Recent studies have demonstrated the efficacy of esketamine in enhancing analgesic profiles and reducing opioid requirements in surgical settings (10).

Target-controlled infusion (TCI) is a modern anesthetic delivery method that maintains constant plasma or effect-site concentrations by adjusting infusion rates based on pharmacokinetic models (11). It is widely used in operating rooms and ICUs to safely administer intravenous anesthesia and analgosedation with agents such as propofol, dexmedetomidine, and opioids (12). The integration of TCI with drugs like esketamine and sufentanil presents a novel anesthetic strategy that may improve perioperative hemodynamic control, reduce postoperative complications, and shorten recovery times.

This study was designed to examine the hemodynamic effects and clinical outcomes of a TCI-based esketamine-sufentanil anesthesia protocol in patients undergoing VATS. By comparing this approach to the conventional sufentanil-propofol regimen, the study sought to determine whether the novel combination could offer superior hemodynamic stability, faster recovery, improved analgesia, and reduced adverse reactions.

## Material and Methods

### Ethics statement

This study was approved by the Ethics Committee of Jinling Hospital, Affiliated Hospital of Medical School, Nanjing University and all study subjects signed an informed consent form.

### Study subjects

Eighty patients admitted to Jinling Hospital, Affiliated Hospital of Medical School, Nanjing University from November 2023 to November 2024 who were proposed to undergo VATS were randomly selected as study subjects. Inclusion criteria: 1) 18∼80 years old; 2) patients without preoperative co-infectious diseases; and 3) patients without a history of alcohol or narcotic drug dependence. Exclusion criteria: 1) patients who received general anesthesia within 1 week; 2) patients who suffered from cardiovascular and cerebrovascular diseases and other basic diseases, with poor control; 3) patients with severe hepatic and renal insufficiency, or psychiatric disorders; 4) patients with long-term use of analgesics; and 5) patients with anesthesia-related drug allergy.

The sample size calculation for this study was based on the expected difference in mean arterial pressure (MAP), the main observation factor. Based on pre-experimental data and previous literature (13), the expected difference in MAP between the TCI group and the conventional empiric infusion group during one-lung ventilation (T3 time point) was approximately 10 mmHg, with a standard deviation of 8 mmHg. Setting α=0.05 (bilateral) and β=0.20 (80% of the test efficacy), the following sample size formula for the comparison of the means of two independent samples was used: n = 2 × [(Zα/2 + Zβ) × σ / δ] ^2^ = 2 × [(1.96 + 0.842) × 8 / 10]^2^ ≅ 2 × 5.02 ≅ 10.04. The corrected formula was: corrected sample size = calculated sample size / (1 - abscission rate). Substituting the results of the above calculations, the required sample size for each group = 10.04 ÷ (1-0.2), which resulted in approximately 12.55, rounded upwards to 13 cases. However, since this initial sample size calculation was itself based on an idealized state, in order to further ensure the stability and reliability of the study and to cope with other possible data biases, we finally determined the sample size of each group to be 40 cases, with a total of 80 patients in the two groups included in the study.

All study subjects were consecutively numbered (No. 1 to 80) in the order of presentation before enrollment. SPSS 26.0 statistical software (IBM, USA) was utilized to generate random numbers, and the patients were randomly divided into the control group and the observation group. Specifically, the SPSS random number generator was used to produce 80 unique random numbers within the range of 1 to 80. These numbers were arranged in ascending order, and each number was assigned to a corresponding patient. According to the sorted sequence, the first 40 numbers were allocated to the control group, while the remaining 40 numbers were assigned to the observation group. After the grouping was completed, the general data such as age, gender, body mass index (BMI), length of surgery, and other general data of the patients in the two groups were statistically analyzed. Control group: aged 60.90 (±9.80) years old, 21 males and 19 females, BMI 21.96 (±4.33) kg/m^2^, average length of operation 4.68 (±1.26) hours, 14 cases using antihypertensive medication. Observation group: aged 61.40 (±10.49) years old, 22 males and 18 females, BMI 22.02 (±4.42) kg/m^2^, average length of operation 4.64 (±0.76) h, 19 cases using antihypertensive medication. There was no significant difference between the general data of the two groups (P>0.05), and they are comparable.

Patients were randomly assigned to different groups without knowing whether they belonged to the control group or the observation group. Healthcare professionals involved in the treatment of patients, data collection, and observation were also unaware of the patient grouping during the study and worked only on the basis of the established intervention protocol. Researchers responsible for data analysis were similarly unaware of patient grouping during data entry and statistical analysis, which was revealed after the analysis was completed. To ensure blinding, the packaging and labeling of the interventions in the control and observation groups were uniformly handled during the design stage of the study protocol to avoid blinding breakage due to differences in appearance. Blinding-related training was provided to all personnel involved in the study to emphasize the importance of blinding in the study and the specific operational specifications.

### Preoperative preparation

All patients were required to fast for 8 h (solid food) and 6 h (clear liquid) before surgery to reduce the risk of gastric reflux and aspiration during anesthesia. For special groups, such as diabetic patients or those with gastrointestinal dysfunction, the fasting time was adjusted appropriately according to the clinical situation, or the blood glucose stability was maintained through intravenous infusion of glucose solution to avoid the occurrence of preoperative hypoglycemia. After the patient entered the operating room, the first step was to establish venous access, using an 18-20G venous needle, and prioritizing the puncture of the thick veins of the upper limbs (e.g., the median elbow vein or the noble vein), in order to ensure the smooth infusion of fluids and medications during the operation. Subsequently, preoxygenation was performed to increase the end-expiratory oxygen concentration (EtO_2_) to more than 90% by continuous oxygen delivery through a mask at an oxygen flow rate of 5-6 L/min for 3-5 min, thus prolonging the safe anaerobic time during tracheal intubation. At the same time, standard monitoring devices were connected, including electrocardiogram (ECG), noninvasive blood pressure (NIBP), and oxygen saturation (SpO_2_), and the patient's baseline vital signs were recorded, which provided a reference basis for subsequent anesthesia management.

### Anesthesia program

For patients in the control group, the conventional anesthesia regimen of sufentanil combined with propofol was applied, and the dosage adjustment of anesthesia drugs mainly relied on the clinical experience of anesthesiologists. During the induction phase of anesthesia, intravenous sufentanil citrate injection (0.2 μg/kg) was administered, and the push time was controlled to be more than 60 s in a bid to reduce the occurrence of adverse reactions, such as respiratory depression and chest wall tonus. Subsequently, propofol lactated injection (2 mg/kg) was given and pushed slowly at a rate of 30-60 s until the patient became unconscious (bispectral index (BIS) decreased to 40-60). According to the patient's hemodynamic changes (e.g., blood pressure, heart rate [HR] fluctuations) and motor responses (e.g., frowning, limb movement) during the operation, the anesthesiologist made an empirical judgment and added propofol or sufentanil as appropriate so as to maintain an appropriate depth of anesthesia.

For patients in the observation group, the anesthesia regimen of esketamine combined with sufentanil based on TCI was used for precise control. During the induction stage, esketamine hydrochloride injection (0.2 mg/kg) was administered intravenously, and the infusion rate was controlled at a speed of more than 60 s, so as to reduce the risk of hallucinations, delirium, and other psychiatric symptoms. Then propofol (2 mg/kg) was given intravenously to play a synergistic effect of sedation, and at the same time, to antagonize sympathomimetic excitation triggered by esketamine. After the patient's consciousness disappeared, intravenous sufentanil (0.3 μg/kg) was injected to complete the analgesia. During the operation, the TCI system was employed to continuously monitor the concentrations of the drugs in the plasma and effector compartment, and the infusion rates of esketamine and propofol were dynamically and precisely adjusted according to the individualization of the patient's age, body weight, and hepatic and renal function. The propofol effector concentration was maintained at 2-4 μg/mL, and the concentration of esketamine was controlled at 100-200 ng/mL. If intraoperative body movement occurred or hemodynamic instability was observed - defined as systolic blood pressure fluctuating by more than 20% from baseline or HR exceeding 100 beats per minute or dropping below 50 beats per minute - the TCI system was programmed to automatically calculate and administer an additional drug dose. Meanwhile, BIS monitoring was used to ensure that the BIS value remained stable between 40 and 60, thereby maintaining an appropriate depth of anesthesia.

### Intraoperative management

Patients in both groups underwent standard intraoperative monitoring, including continuous ECG, invasive/non-invasive blood pressure, SpO_2_, end-expiratory carbon dioxide (PETCO_2_), and BIS values. In case of hypotension (MAP<65 mmHg), the first step was to accelerate crystalloid infusion and use vasoactive drugs (e.g., phenylephrine) if necessary; in case of hypertension (MAP>20% of the baseline value), antihypertensive drugs (e.g., uradil) were administered after ruling out too shallow anesthesia.

### Postoperative analgesic program

The patients in the observation group were connected to a patient-controlled intravenous analgesia (PCIA) pump, and the formula used was 1 mg/kg esketamine compounded with 1.5 μg/kg sufentanil, plus 0.6 mg remosetron, diluted to 100 mL with 0.9% sodium chloride injection. The parameters of the PCIA pump were set as the base rate of 2 mL/h, the self-control dose of 1.5 mL, and the locking time of 15 min. The program referred to the synergistic analgesic effect of esketamine and sufentanil and could reduce the dosage and adverse effects of each single drug. In the control group, the PCIA formula was 2 μg/kg of sufentanil and 0.6 mg of ramosetron in 100 mL of 0.9% sodium chloride injection, and the parameters were the same as those in the observation group.

### Observation indicators

#### Hemodynamic indicators

The following indices were compared between the two groups of patients at the time points before anesthesia (T0), after induction of anesthesia (T1), 30 min of anesthesia (T2), and at the end of surgery (T3). i) MAP: it reflects the average pressure of blood against the walls of blood vessels in the circulatory system and is an important indicator for monitoring circulatory function; ii) HR: the number of times the heart beats per minute, a basic indicator of the state of cardiac activity; iii) central venous pressure (CVP): pressure (mmHg) in the right atrium or adjacent large vein, reflecting blood volume and right heart function; iv) stroke volume (SV): volume of blood ejected per ventricular contraction (mL/beat), calculated from cardiac output/HR, which reflects myocardial contractility, and decreases may be due to hypovolemia, cardiac insufficiency, or increased afterload; v) cardiac output (CO): blood volume pumped by the heart per minute (L/min)=SV × HR, which is a key indicator of systemic oxygen supply, with a normal value of 4-8 L/min; vi) systemic vascular resistance (SVR): resistance to blood flow (dyne-s/cm^5^) in the vessels of the body circulation, elevated in vasoconstriction (e.g., stress, hypothermia) and decreased in vasodilatation (e.g., anesthetic drugs, sepsis); vii) SpO_2_: the percentage of oxygenated hemoglobin to total hemoglobin in the blood, which is an important indicator to assess the oxygenation of the body.

#### Length of stay in the post-anesthesia care unit (PACU) and time of awakening

The PACU records the time from when the patient enters the post-anesthesia recovery room at the end of the procedure to when he or she leaves the recovery room. During this time, the patient needs to be closely monitored and observed in the recovery room to ensure that his/her vital signs are stable and no complications occur. Wake-up time refers to the time from the beginning of the anesthesia state to the time when the patient has fully regained consciousness and is able to make appropriate movements or answer simple questions according to instructions.

#### Ramsay sedation score (RSS)

The RSS was evaluated at T0, T1, T2, and T3 (14). It categorizes the degree of sedation into six grades by observing the physiological responses and behavioral manifestations of the patients: 1 for irritable and restless; 2 for awake and quiet and cooperative; 3 for sleepy and responsive to commands; 4 for light sleepy state, which can be rapidly awakened; 5 for asleep, unresponsive to calls; 6 for deep sleep state, difficult to awaken.

#### Postoperative pain

A visual analog scale (VAS) (15) was employed to assess the pain at 2, 24, and 48 h postoperatively. A 10-cm horizontal line was drawn on a piece of paper, with 0 mm at one end of the line indicating “no pain at all” and 10 mm at the other end indicating “extreme pain”.

#### Incidence of adverse reactions

The occurrence of adverse reactions such as nausea, vomiting, dizziness and headache, hypotension, and drowsiness was recorded in both groups after surgery.

### Statistical analysis

SPSS 26.0 software was utilized for statistical analysis of the data. Before formal statistical inference, data were assessed for normal distribution by the Shapiro-Wilk test when the sample size was n≤50, and by the Kolmogorov-Smirnov test when n>50. The results showed that all measures in this study met normal distribution (P>0.05) criteria and there were no skewed distribution data, so parametric tests were used. The results of the measurement data are reported as means±SD, and *t*-test was applied for comparison between the two groups. Repeated measures ANOVA was employed to analyze tests used for comparison of multiple time points. Categorical data are reported as rates or percentages, and comparisons were made using the chi-squared test. P<0.05 (two-sided) was considered statistically significant.

## Results

### Hemodynamic indicators

There were no significant intergroup differences in baseline hemodynamic parameters, including MAP, HR, CVP, SV, CO, SVR, and SpO_2_ before anesthesia induction (T0) (P>0.05). From T1 to T3, the patients in both groups showed a notable decrease in MAP, HR, SV, CO, and SpO_2_ compared with that of T0 (P<0.05). These declines were consistently less pronounced in the observation group relative to the control group (P<0.05). At T3, MAP, SV, and CO in the observation group were significantly higher than those in the control group (P<0.05) with reduced hemodynamic variability. In addition, CVP and SVR intraoperative fluctuations were more stable in the observation group, and SpO_2_ remained consistently higher than those in the control group (P<0.05) (Table 1).

**Table 1:** Hemodynamic indicators of the control group (conventional empiric anesthesia) and the observation group (target-controlled infusion).

Indicators	Time	Control group	Observation group	F	P
		(n=40)	(n=40)		
MAP (mmHg)	T0	117.05±3.43	117.25±3.48	935.720 (time), 123.670 (between groups), 70.238 (interaction)	<0.001 (time), <0.001 (between groups), <0.001 (interaction)
	T1	97.23±4.99^b^	107.70±3.36^ab^		
	T2	93.60±4.92^bc^	102.63±3.29^abc^		
	T3	90.15±4.53^bcd^	101.88±3.28^abcd^		
HR (beats/min)	T0	117.00±2.83	117.05±4.06	935.720 (time), 123.670 (between groups), 70.238 (interaction)	<0.001 (time), <0.001 (between groups), <0.001 (interaction)
	T1	107.58±2.60^b^	110.70±4.48^ab^		
	T2	77.18±3.43^bc^	87.60±4.18^abc^		
	T3	71.93±3.72^bcd^	85.33±4.25^abcd^		
CVP (mmHg)	T0	5.07±0.72	5.06±0.77	7758.884 (time), 81.164 (between groups), 204.254 (interaction)	<0.001 (time), <0.001 (between groups), <0.001 (interaction)
	T1	3.68±0.75^b^	4.32±0.75^ab^		
	T2	4.16±0.70^bc^	4.56±0.74^bc^		
	T3	6.08±0.69^bcd^	5.71±0.76^bcd^		
SV (mL/beat)	T0	74.33±10.54	74.80±10.66	103.588 (time), 8.779 (between groups), 14.174 (interaction)	0.004 (time), 0.001 (between groups), <0.001 (interaction)
	T1	59.80±10.43^b^	69.55±10.17^ab^		
	T2	65.30±10.56^bc^	72.08±10.59^abc^		
	T3	57.90±9.66^bcd^	66.00±10.15^abcd^		
CO (L/min)	T0	5.62±1.01	5.65±1.05	276.725 (time), 5.941 (between groups), 25.381 (interaction)	0.017 (time), 0.015 (between groups), <0.001 (interaction)
	T1	4.16±1.04^b^	4.96±0.96^ab^		
	T2	4.56±0.98^bc^	5.20±1.06^abc^		
	T3	4.02±0.99^bcd^	4.67±0.94^abcd^		
SVR (dyne-s/cm^5^)	T0	1202.4±153.25	1202.55±144.96	4859.438 (time), 0.577 (between groups), 947.505 (interaction)	0.450 (time), 0.822 (between groups), <0.001 (interaction)
	T1	899.55±154.78^b^	1052.55±144.96^b^		
	T2	1103.40±153.25^bc^	1153.55±144.96^bc^		
	T3	1300.83±163.66^bcd^	1199.55±144.96^abcd^		
SpO_2_ (%)	T0	97.98±1.27	98.05±1.43	1197.787 (time), 56.014 (between groups), 34.760 (interaction)	<0.001 (time), <0.001 (between groups), <0.001 (interaction)
	T1	93.88±1.42^b^	95.65±1.55^ab^		
	T2	89.53±1.41^bc^	92.65±1.56^abc^		
	T3	87.38±1.39^bcd^	90.23±1.76^abcd^		

Data are reported as means and SD. ^a^P<0.05 *vs* the control group at the same time point. ^b^P<0.05 *vs* the same group at T0. ^c^<0.05 *vs* the same group at T1. ^d^P<0.05 *vs* the same group at T2. Statistical analysis was performed using repeated measures ANOVA. MAP: mean arterial pressure; HR: heart rate; CVP: central venous pressure; SV: stroke volume; CO: cardiac output; SRV: systemic vascular resistance; SpO_2_: oxygen saturation; T0: before anesthesia; T1: after induction of anesthesia; T2: 30 min of anesthesia; T3: at the end of surgery.

### Length of stay in the PACU and time of awakening

The length of PACU and the awakening time of patients in the observation group were lower than in the control group (P<0.05) (Table 2).

**Table 2:** Length of stay in the post-anesthesia care unit (PACU) and awakening time after anesthesia in the control group (conventional empiric anesthesia) and the observation group (target-controlled infusion).

	Time in PACU (h)	Wake-up time (h)
Control (n=40)	3.73±0.64	10.61±0.96
Observation (n=40)	2.86±0.71	8.49±0.93
t	5.720	10.085
P	<0.01	<0.01

Data are reported as means and SD. Independent samples t-test was used for inter-group comparisons.

### RSS

There was no notable difference between the RSS of the two groups at T0 (P>0.05). At T1 to T3, the RSS of both groups increased, and the scores of the observation group were noticeably higher than the control group at the T2 and T3 time points (P<0.01) (Table 3).

**Table 3:** Ramsay sedation scores for the control group (conventional empiric anesthesia) and the observation group (target-controlled infusion).

Groups	T0	T1	T2	T3
Control (n=40)	2.08±0.83	2.50±0.72^b^	2.10±1.06^c^	1.95±0.64^bcd^
Observation (n=40)	2.03±0.36	2.73±0.60^b^	3.50±0.78^abc^	2.73±0.45^bcd^
F	20.769 (time), 35.548 (between groups), 19.911 (interaction)			
P	<0.001 (time), <0.001 (between groups), <0.001 (interaction)			

Data are reported as means and SD. Data analysis was conducted using repeated measures ANOVA. ^a^P<0.05 *vs* the control group at the same time point. ^b^P<0.05 *vs* the same group at T0. ^c^P<0.05 *vs* the same group at T1. ^d^P<0.05 *vs* the same group at T2. T0: before anesthesia; T1: after induction of anesthesia; T2: 30 min of anesthesia; T3: at the end of surgery.

### Postoperative pain

Relative to the control group, the observation group tended to report lower VAS pain scores at 2, 24, and 48 h after surgery. The differences became statistically meaningful at 24 and 48 h postoperatively (P<0.05) (Table 4).

**Table 4:** Comparison of VAS pain scores between the control group (conventional empiric anesthesia) and the observation group (target-controlled infusion).

Groups	2 h after surgery	24 h after surgery	48 h after surgery
Control group (n=40)	1.48±0.60	5.53±0.55^b^	4.43±0.50^c^
Observation group (n=40)	1.43±0.50	4.48±0.64^ab^	2.40±0.71^abc^
F	3407.141 (time), 68.473 (between groups), 290.283 (interaction)		
P	<0.001 (time), <0.001 (between groups), <0.001 (interaction)		

Data are reported as means and SD. ^a^P<0.05 *vs* the control group at the same time point. ^b^P<0.05 *vs* the same group at 2 h postoperatively. ^c^P<0.05 *vs* the same group at 24 h postoperatively. Data analysis was conducted using repeated measures ANOVA.

### Incidence of adverse reactions

Patients in the observation group experienced fewer postoperative adverse events than those in the control group (P<0.05) (Table 5).

**Table 5:** Comparison of adverse reactions between the two groups.

	Nauseating	Vomiting	Headache	Low blood pressure	Drowsiness	Total incidence
Control group (n=40)	4 (15.00)	0 (0.00)	5 (12.50)	1 (2.50)	4 (10.00)	14 (35.00)
Observation group (n=40)	2 (5.00)	0 (0.00)	1 (2.50)	0 (0.00)	2 (5.00)	5 (12.50)
X^2^	5.591					
P	0.018					

Data are reported as number and percentage. Chi-squared test was used for inter-group comparisons.

## Discussion

Due to its minimally invasive nature, reduced postoperative pain, and faster recovery, VATS has become a preferred approach over traditional open thoracotomy (16). However, the use of one-lung ventilation combined with intrathoracic manipulations in VATS can trigger intraoperative hypoxemia and hemodynamic variations, increasing the risk of perioperative myocardial injury (17). The findings of this study confirmed that a TCI anesthesia protocol using esketamine combined with sufentanil offered superior hemodynamic control, faster recovery, and improved tolerability compared to the conventional sufentanil-propofol regimen.

The enhanced hemodynamic stability in the TCI group, particularly the higher MAP, SV, and CO at T3, may be attributed to the synergistic effects of esketamine and sufentanil. Esketamine exerts sympathomimetic effects by inhibiting catecholamine reuptake, thereby mitigating the hypotension often induced by opioids like sufentanil (18,19). This mechanism is supported by a recent study by Wang et al. (20), which reported that esketamine infusion during major surgery attenuated opioid-induced hypotension while maintaining adequate analgesia. Additionally, as a clinically-available and widely-used computer-controlled method of drug administration (21), TCI allows for stable plasma concentrations of esketamine and sufentanil, avoiding the bolus-related peaks and troughs that are typical of manually adjusted infusion regimens, further minimizing cardiovascular fluctuations. In contrast, propofol - used in the control group - has well-documented vasodilatory and myocardial depressive effects (22,23), which likely contributed to the greater hemodynamic instability observed.

Moreover, faster postoperative recovery in the esketamine group can also be ascribed to both the pharmacokinetics of the drug and the benefits of TCI-based dosing. Esketamine has a relatively short distribution half-life and rapid systemic clearance (24), resulting in less drug accumulation compared to lipophilic agents like propofol. The ability of TCI systems to maintain narrow target concentrations minimizes excessive sedation and allows for quicker return of consciousness. Furthermore, the superior postoperative analgesia seen in this group may stem from the dual mechanism of action; esketamine provides central desensitization through NMDA receptor blockade (25), while sufentanil acts peripherally and centrally via μ-opioid receptors (26). The synergistic analgesic effect of the combination allows for dose reduction of each agent, enhancing pain control while mitigating opioid-related adverse events such as nausea and somnolence. Notably, a recent study by Qi et al. (27) further confirms the comprehensive perioperative benefits of esketamine: their findings demonstrate that esketamine not only reduces the incidence of postoperative nausea and vomiting but also decreases perioperative opioid consumption and improves postoperative pain scores. A study by Mion (28) has also proven that esketamine permits roughly 50% lower dosing for anesthesia and pain relief compared to racemic ketamine, along with more rapid elimination and quicker recovery.

Additionally, the lower incidence of adverse effects in the TCI group further supports the safety profile of this approach. Unlike traditional ketamine, esketamine has a lower risk of psychomimetic effects due to its higher potency and cleaner receptor binding (29). Esketamine is known to preserve spontaneous breathing (30) and upper airway reflexes (31), in contrast to the respiratory depressant effects of propofol and high-dose opioids. This may explain the lower rates of desaturation and hypotension observed in our study. Moreover, the TCI algorithm minimizes sudden surges in drug concentration, reducing the likelihood of acute side effects such as dizziness, vomiting, and hemodynamic suppression (32). Our study also provided further support for the conclusions drawn by Qi et al. (27), revealing the unique value of esketamine in reducing the common and patient-distressing complication of nausea and vomiting, thereby highlighting its favorable safety profile. These benefits are especially relevant in thoracic anesthesia, where even minor respiratory compromise can lead to serious complications.

To sum up, the combination of esketamine and sufentanil delivered via TCI offered a clinically advantageous strategy for VATS. It achieved greater hemodynamic stability, more consistent sedation, enhanced analgesia, and fewer complications when compared to conventional methods. Building upon previous research confirming esketamine's efficacy in reducing postoperative nausea and vomiting and opioid requirements, our study further elucidated its contribution to achieving precise anesthesia, optimizing hemodynamic management, and promoting postoperative recovery through the application of TCI technology. These results suggest that precision-guided anesthesia using individualized pharmacokinetic models may be a promising direction in thoracic surgery, particularly for procedures with high physiological stress, such as thoracoscopic operations. However, this study was limited by its single-center design, relatively small sample size, and lack of long-term postoperative outcome data. In addition, patient variability in pharmacokinetic response was not fully explored, which may affect generalizability. Further multicenter trials with larger cohorts and extended follow-up are warranted to confirm these findings, optimize TCI protocols, and assess cost-effectiveness and patient-reported outcomes in broader surgical populations.

## Data Availability

All data generated or analyzed during this study are included in this published article.
